# Direct Detection of Circularly Polarized Light Using Chiral Copper Chloride–Carbon Nanotube Heterostructures

**DOI:** 10.1021/acsnano.1c01134

**Published:** 2021-04-06

**Authors:** Ji Hao, Haipeng Lu, Lingling Mao, Xihan Chen, Matthew C. Beard, Jeffrey L. Blackburn

**Affiliations:** † Chemistry & Nanoscience Center, National Renewable Energy Laboratory, Golden, Colorado 80401, United States; ■ Materials Science Center, National Renewable Energy Laboratory, Golden, Colorado 80401, United States; ‡ Department of Chemistry, The Hong Kong University of Science and Technology, Clear Water Bay, Kowloon, Hong Kong, China (SAR); § Materials Department and Materials Research Laboratory University of California, Santa Barbara, California 93106, United States

**Keywords:** chiral organic−inorganic hybrid materials, chiral copper chloride, carbon nanotube, circularly polarized light detection, heterojunction, optoelectronics

## Abstract

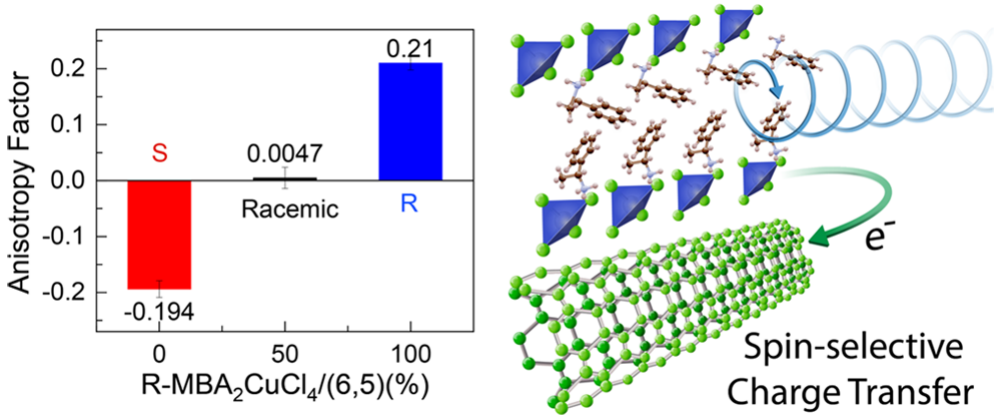

The emergent properties of chiral organic–inorganic hybrid materials offer opportunities in spin-dependent optoelectronic devices. One of the most promising applications where spin, charge, and light are strongly coupled is circularly polarized light (CPL) detection. However, the performance of state-of-the-art CPL detectors using chiral hybrid metal halide semiconductors is still limited by the low anisotropy factor, poor conductivity, and limited photoresponsivity. Here, we synthesize 0D chiral copper chloride hybrids, templated by chiral methylbenzylammonium (*R*/*S*-MBA), *i.e.*, (*R*-/*S*-MBA)_2_CuCl_4_, that display circular dichroism for the ligand-to-metal charge transfer transition with an absorption anisotropy factor (*g*_CD_) among the largest reported for chiral metal halide semiconductor hybrids. To circumvent the poor conductivity of the unpercolated inorganic framework of this chiral absorber, we develop a direct CPL detector that utilizes a heterojunction between the chiral (MBA)_2_CuCl_4_ absorber layer and a semiconducting single-walled carbon nanotube (s-SWCNT) transport channel. Our chiral heterostructure shows high photoresponsivity of 452 A/W, a competitive anisotropy factor (*g*_res_) of up to 0.21, a current response in microamperes, and low working voltage down to 0.01 V. Our results clearly demonstrate a useful strategy toward high-performance chiral optoelectronic devices, where a nanoscale heterostructure enables direct CPL detection even for highly insulating chiral materials.

## Introduction

Detection of circularly polarized light (CPL) is of great importance for the development of various optical technologies, including optical imaging,^1,2^ remote sensing,^3^ quantum computing^4,5^ and information processing and communication.^6,7^ Conventional optical detectors require coupling with optical polarizers to detect CPL, which often limits their sensitivity and resolution. In contrast, direct detection of the polarization state of CPL can be achieved in chiral systems that display circular dichroism (CD), *i.e.*, distinct absorption coefficients for left- and right-handed CPL.^8−14^ The challenge for integrating efficient chiral absorbers into CPL detectors is effectively transducing the optical CD into sufficiently large electrical signals and amplifying the discrimination (anisotropy factor) between the different photon helicities.

The development of chiral organic/inorganic metal halide semiconductors (MHS)^9,15−18^ is a very promising solution to realize the direct detection of CPL^19^ because of their chiroptical activity and superior optoelectronic properties.^20^ Additionally, charge transport in chiral MHS can be highly dependent on the carrier spin sense *via* the chiral-induced spin-selectivity (CISS) mechanism,^17,18,21^ thus providing additional tuning parameters to distinguish the polarization state of CPLs (as CPL carries +1> or −1> angular momentum). A series of reports have recently demonstrated direct CPL detection using chiral-MHS. For instance, in 2020, Chen *et al.*(9) and Wang *et al.*(13) separately demonstrated direct CPL photodetectors using 1D and quasi-2D chiral perovskite semiconductors within simple two-terminal electronic device structures. These promising initial demonstrations can still be improved upon, as these architectures require high operating voltages (10–20 V) and produce relatively low output current (∼pA). Very recently, Ishii *et al.* improved upon these initial studies, demonstrating a high CPL anisotropy factor and current density up to 0.28 mA/cm^2^ for a vertical device with a 1D chiral MHS.^22^

Despite their high CD, many low-dimensional chiral MHS suffer from low, or even negligible, conductivity and/or photoconductivity. Thus, broader development of CPL detectors will require strategies to circumvent this poor transport. Ma *et al.*(11) demonstrated that a heterojunction between a 2D chiral perovskite and 2D MoS_2_ could be used as a CPL photodetector, but the photocurrent (tens of pA) and anisotropy factor (*g*_res_, defined below) were still modest. Improvements to CPL detection anisotropy, current amplitude, and operating voltages should benefit from the development of (1) chiral-MHS with larger intrinsic CD and (2) heterostructures with high-mobility semiconductors that facilitate spin-dependent photoinduced charge transfer.

Here, we demonstrate that we can simultaneously address these two challenges with the following strategies. We first synthesize a series of 0D chiral copper chloride hybrids, templated by methylbenzylammonium enantiomers (*R*/*S*-MBA), *i.e.*, (*R*-/*S*-MBA)_2_CuCl_4_. Transmission CD measurements show that chiral (*R*-/*S*-MBA)_2_CuCl_4_ thin films display a much larger optical anisotropy compared to previously reported 2D and 1D chiral-MHS.^9,16−18,22^ We combine these insulating chiral copper chloride hybrids with electronically coupled semiconducting single-walled carbon nanotube (s-SWCNT) networks to form MBA_2_CuCl_4_/SWCNT heterostructures that can readily transduce incident CPL into large electrical signals with a high anisotropy factor. This heterostructure utilizes the effect of spin-selective interfacial charge transfer^23^ to capitalize upon the large chiroptical response of (*R*-/*S*-MBA)_2_CuCl_4_ absorber layers and the superior carrier transport property of the SWCNT transport channel. Circularly polarized photons are first absorbed by chiral MBA_2_CuCl_4_, which undergoes an ultrafast electron transfer to the SWCNT layer, generating sensitive polarization-dependent photoresponsivity. We demonstrate a high photoresponsivity of 452 A/W, a microampere level photocurrent, a low working voltage of 0.01 V, and a high anisotropy factor (*g*_res_) of up to 0.21. These performance characteristics compare well with those of reported CPL detectors using chiral 1D and 2D hybrid semiconductors only,^9,13,22,24^ demonstrating a promising heterojunction-based strategy for high-performance chiral optoelectronic devices that utilize spin-selective charge transfer at rationally designed nanoscale interfaces.

## Results and Discussion

### Crystal Structure of Chiral Copper Chloride Hybrids

**Figure 1:**
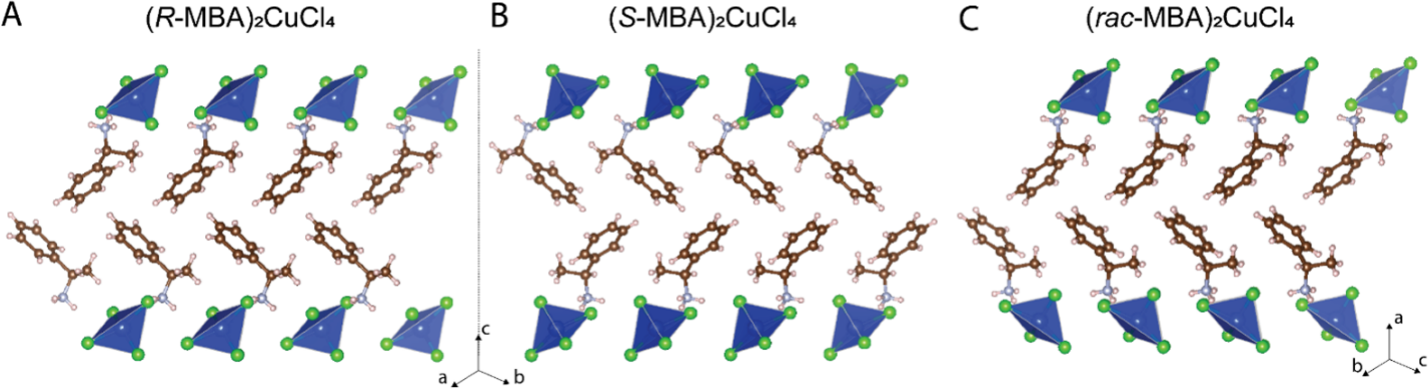
Crystal structure of (A) (*R-*MBA)_2_CuCl_4_, (B) (*S-*MBA)_2_CuCl_4_ from the side view along the *c* direction, and (C) racemic phase (*rac-*MBA)_2_CuCl_4_ from the side view along the *a* direction.

Single crystals of chiral (*R-*/*S-*MBA)_2_CuCl_4_ and the racemic phase (*rac-*MBA)_2_CuCl_4_ were grown from concentrated isopropyl alcohol solutions containing stoichiometric amounts of MBACl and CuCl_2_·2H_2_O. Cooling to room temperature yielded thin green platelets of (MBA)_2_CuCl_4_. Crystallographic data and structure refinement information are summarized in Table S1. Similar to previously reported chiral Pb–I^8^ and Sn–I hybrids,^18^ the chiral (*R-*MBA)_2_CuCl_4_ and (*S-*MBA)_2_CuCl_4_ both crystallize in a chiral space group *C*2, while the racemic phase (*rac-*MBA)_2_CuCl_4_ crystallizes in the achiral orthorhombic space group *Aea*2. The crystal structures appear to be a layered lamellar structure (Figure 1), with a layer of disconnected CuCl_4_ tetrahedra and a bilayer of organic MBA cations. The overall crystal structures of (*R-*/*S-*MBA)_2_CuCl_4_ and (*rac-*MBA)_2_CuCl_4_ are very similar. However, the crystallographic packing direction is along the *c* direction for (*R-*/*S-*MBA)_2_CuCl_4_, whereas it is along the *a* direction for (*rac*-MBA)_2_CuCl_4_. Additionally, the unit cell size of (*rac*-MBA)_2_CuCl_4_ is around twice that of the (*R-*/*S-*MBA)_2_CuCl_4_ compounds.

**Figure 2:**
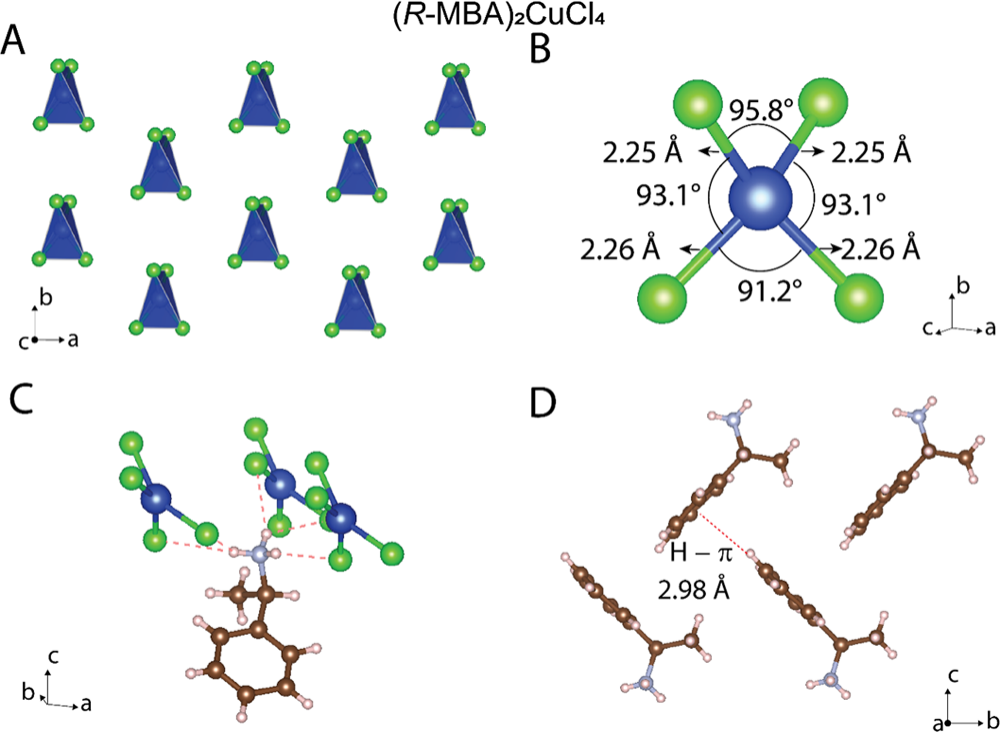
(A) Crystal structure of (*R-*MBA)_2_CuCl_4_ from the top-down view (organic cations are omitted for clarity). (B) Bond length and bond angles with a CuCl_4_ tetrahedron. (C) Hydrogen bonds between the inorganic CuCl_4_ and organic MBA cations. (D) Crystal packing arrangement for organic MBA cations showing the C–H···π interactions (inorganic CuCl_4_ are omitted for clarity).

It is informative to compare the structure of the MBA_2_CuCl_4_ compounds to an achiral analogue, phenylethylammonium copper chloride (PEA_2_CuCl_4_), in which the crystal structure consists of layered corner-shared CuCl_6_ octahedra (with Jahn–Teller distortion).^25^ In contrast, with MBA^+^ as the templating cations, the inorganic Cu–Cl forms distorted disconnected CuCl_4_ tetrahedra. These distorted CuCl_4_ tetrahedra can be conceptionally derived from the corner-sharing Jahn–Teller-distorted CuCl_6_ octahedra in two steps: (1) the elongated Cu–Cl bonds become much longer so that the corner-sharing octahedra become disconnected; (2) the square planar CuCl_4_^2–^ ions are further distorted to a tetrahedron geometry (Figure 2A). For example, in (*R*-MBA)_2_CuCl_4_, each CuCl_4_ tetrahedron consists of two Cu–Cl bonds with 2.25 Å length and two with 2.26 Å length (Figure 2B). The Cl–Cu–Cl bond angles within each tetrahedron range from 91.2 to 95.8°, all of which are significantly modified from an ideal undistorted tetrahedron (109.5°). Such large CuCl_4_ tetrahedral distortion is likely a synergic effect from both a Jahn–Teller distortion in the inorganic CuCl and hydrogen bonds between the inorganic CuCl_4_^2–^ and organic MBA^+^ cations. Figure 2C shows that the NH_3_ headgroup in each MBA^+^ cation forms five hydrogen bonds with the inorganic CuCl_4_ layer. In addition, organic MBA^+^ cations also interact with adjacent cations through C–H···π interactions (2.98 Å, Figure 2D). These noncovalent interactions thus stabilize the three-dimensional crystal packing in the MBA_2_CuCl_4_ hybrids. It is worth noting that these MBA_2_CuCl_4_ compounds were previously reported and studied for their biological activities,^26^ but their optical properties, especially how their crystal structure impacts the chiroptical response, had never been investigated.

### Optical Properties of (MBA)_2_CuCl_4_ and (PEA)_2_CuCl_4_

**Figure 3:**
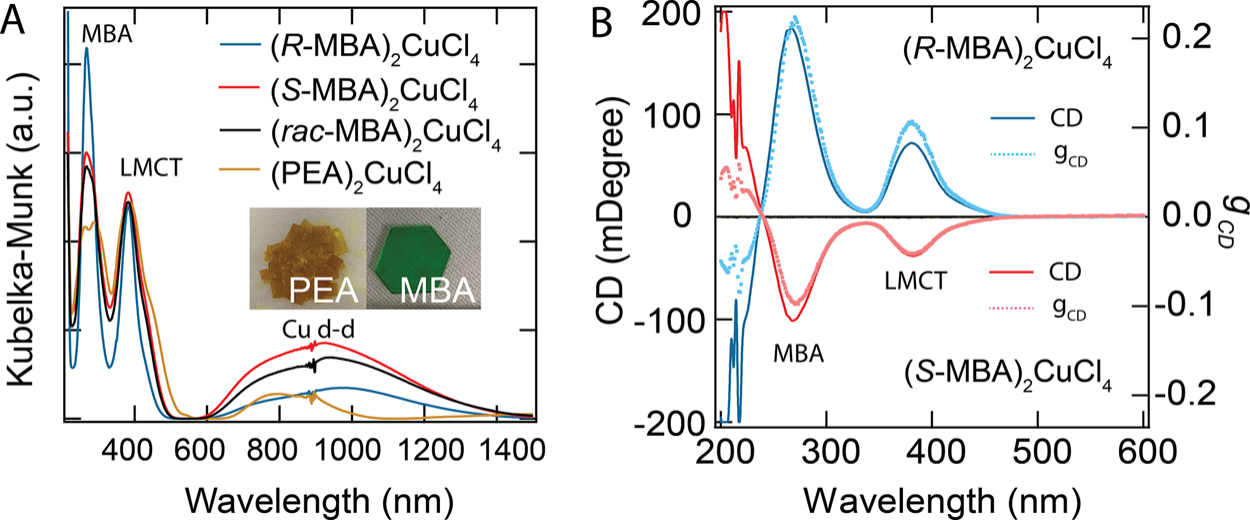
Linear UV–vis–NIR absorption spectra (A) and CD spectra (B) for (*R-*/*S-*/*rac*-MBA)_2_CuCl_4_ and (PEA)_2_CuCl_4_ compounds. Inset in (A) shows photographs of (PEA)_2_CuCl_4_ and (*R*-MBA)_2_CuCl_4_ crystals. In panel (B), the CD signal is transformed to an anisotropy factor, *g*_CD_, according to eq 2.

Optical properties of the as-synthesized (MBA)_2_CuCl_4_ and (PEA)_2_CuCl_4_ crystals are further characterized by linear absorption and CD spectroscopy. Linear optical spectra were measured by diffuse reflectance spectroscopy, and the absorption spectra of powders were obtained by converting reflectance to absorption using the Kubelka–Munk equation. In strong contrast to the distinct differences in physical appearance between (MBA)_2_CuCl_4_ and (PEA)_2_CuCl_4_ crystals (Figure 3A, inset), their UV–vis–NIR absorption spectra appear to be quite similar. Both (MBA)_2_CuCl_4_ and (PEA)_2_CuCl_4_ display two strong UV absorption peaks (270 and 383 nm) and a broad vis–NIR band. However, the vis–NIR bands above 580 nm for (MBA)_2_CuCl_4_ compounds are significantly broader compared to the (PEA)_2_CuCl_4_ compound. The vis–NIR band in the (PEA)_2_CuCl_4_ compound covers from 650 to 1085 nm (0.76 eV range), while that of the (MBA)_2_CuCl_4_ compound covers from 580 to 1500 nm (1.31 eV range). The absorption peak at 270 nm is associated with the π → π* transition of MBA^+^, while the peak at 383 nm and the vis–NIR band are assigned to the ligand-to-metal charge transfer (LMCT) transition and the Cu d–d transitions, respectively, similar to what is reported for other layered Cu–Cl hybrid systems.^27^ Therefore, when switching from PEA^+^ to MBA^+^ as the templating cations, the LMCT band gap of these copper chloride hybrids essentially remains the same, while the Cu d–d transitions become more dispersed. The more dispersed Cu d–d transitions are thus consistent with the higher degree of the structural distortion of the inorganic CuCl_4_ sublattice in the (MBA)_2_CuCl_4_ compounds.

To probe the chiroptical response in these chiral copper chloride hybrids, we prepared polycrystalline thin films by spin-casting a DMF solution of the corresponding crystals. Thin films of (*R-*MBA)_2_CuCl_4_ and (*S-*MBA)_2_CuCl_4_ display distinct bisignate CD peaks from 200 to 470 nm, while (*rac-*MBA)_2_CuCl_4_ and (PEA)_2_CuCl_4_ show no CD. The CD peaks at 380 nm correlate well with their linear absorption peaks (at 383 nm), indicating that the optical LMCT transitions associated with the inorganic CuCl_4_ sublattice gain a chiroptical response resulting from the incorporation of the chiral MBA^+^ cations. This chiroptical transfer from organic sublattice to the inorganic sublattice is consistent with our previous observations in chiral MBA_2_PbI_4_ and MBA_2_SnI_4_ compounds,^17,18^ suggesting that the approach of combining chiral organic cations and inorganic metal halides appears to be a universal strategy to prepare chiral hybrid semiconductors. The chiroptical response mainly stems from the nonzero electric and magnetic transition dipole moments in a chiral space group, giving a nonzero rotational strength (*R*) as
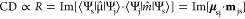
1where Ψ_s_ and Ψ_j_ are the wave functions of the ground state and excited state and **μ**_sj_ and **m**_js_ are the electric and magnetic transition dipole moments, respectively. Since (*R*-/*S*-MBA)_2_CuCl_4_ crystallizes in a noncentrosymmetric chiral space group, both **μ**_sj_ and **m**_js_ ≠ 0, and the compounds should display CD.

It is also worth noting that the chiroptical response from the inorganic CuCl_4_ sublattice in (*R-/S-*MBA)_2_CuCl_4_ thin films shows much larger CD strength (in mdeg) compared to the chiral MBA_2_PbI_4_ and MBA_2_SnI_4_ compounds with similar absorbance (in o.d.). The optical anisotropy factor, *g*_CD_,^28^ is used to normalize the chiroptical response with respect to absorbance:
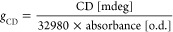
2The *g*_CD_ factor of the LMCT peak at 380 nm is ∼0.1 for (*R-*MBA)_2_CuCl_4_ thin film (Figure 3B), which is around 3–5 times larger than that of 1D MBAPbI_3_ (∼0.02)^9^ and 1D NEAPbI_3_ (∼0.04)^22^ and around 2 orders of magnitude larger than that of 2D MBA_2_PbI_4_ films (∼10^–3^).^17^ Therefore, our results indicate that lower-dimensional chiral metal halide hybrids appear to display an intrinsic chiroptical response larger than that of higher-dimensional chiral metal halide hybrids (see also Table S2). It is currently unclear why the *g*_CD_ factor is higher for the *R*-compound relative to that for the *S*-compound. It is possible that the samples possess differences in morphology, as it is not unusual in nature that enantiomers crystallize with different rates. Even a small difference in dissolution/recrystallization kinetics can lead to a macroscopic difference in the film morphology/thickness. However, both our CD and CPL detection measurements (*vide infra*) indicate that *R*-films consistently possess higher asymmetric factor than *S*-films.

### MBA_2_CuCl_4_/SWCNT Heterojunctions

Although (*R-/S-*MBA)_2_CuCl_4_ thin films possess good chiroptical activity to distinguish the polarization state of CPL, it is electrically insulating (Figure S1), due to the lack of connectivity between isolated CuCl_4_ tetrahedra. An appropriately designed interface with a highly conductive transport material is therefore needed to effectively transduce photons absorbed by MBA_2_CuCl_4_ into electrical current. To address this, we combine chiral MBA_2_CuCl_4_ with highly enriched (6,5) semiconducting SWCNTs to form the active layer for CPL detection. Such highly enriched s-SWCNT networks make high-mobility field-effect transistor (FET) channels^29^ and have been used to efficiently collect photogenerated charges from a variety of organic,^30^ inorganic,^31^ and hybrid metal halide^32^ semiconductors. The chiral MBA_2_CuCl_4_/SWCNT heterojunctions are prepared by spin-coating the chiral MBA_2_CuCl_4_ materials on top of (6,5) SWCNT networks, either on quartz substrates for optical characterization or on bottom-gated and bottom-contacted FET architectures for electrical characterization. Unless otherwise noted, the heterojunctions have a total thickness of 50 nm, with the SWCNT layer being 10 nm thick.

**Figure 4:**
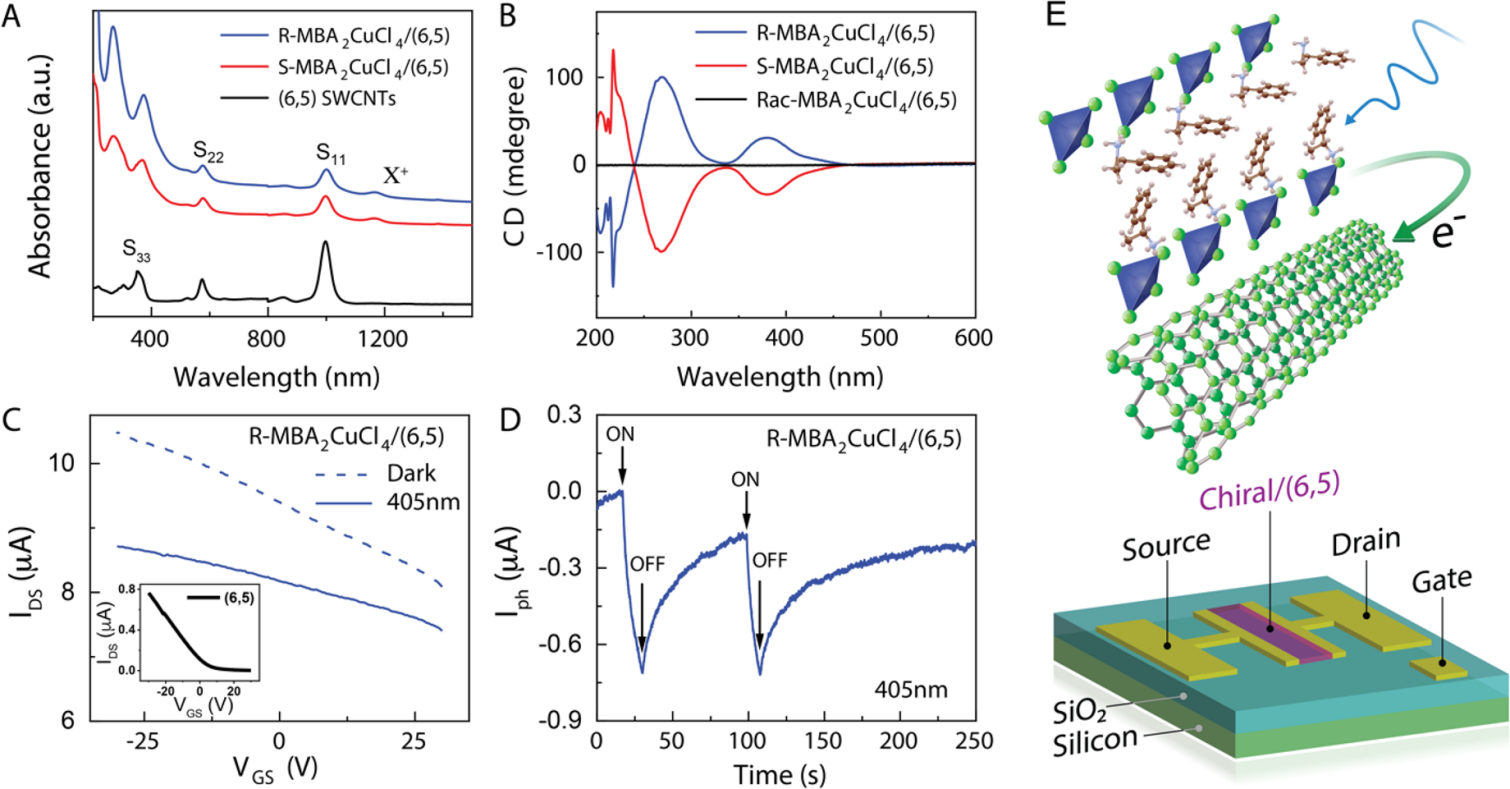
Photoexcited charge transfer in bilayer chiral MBA_2_CuCl_4_/(6,5) SWCNT heterojunctions. (A) Absorption spectra of a typical (6,5) SWNT network, *R*-MBA_2_CuCl_4_/SWCNT heterojunction, and *S*-MBA_2_CuCl_4_/SWCNT heterojunction. Spectra are offset for clarity. (B) CD spectra of *R*- and *S*-MBA_2_CuCl_4_/(6,5) SWCNT heterojunction and (*rac*-MBA)_2_CuCl_4_/(6,5) SWCNT heterojunction. (C) FET transfer curve of *R*-MBA_2_CuCl_4_/(6,5) SWCNT heterojunction in the nitrogen-filled glovebox under *V*_DS_ = 0.1 V. The transfer curve of (6,5) SWCNTs under *V*_DS_ = 3 V is shown in the inset. (D) Negative photocurrent response of (*R*-MBA)_2_CuCl_4_/SWCNT heterojunction under *V*_DS_ = 0.1 V as a function of time as the 405 nm laser (1.1 × 10^–3^ mW/cm^2^) is successively turned on and off. (E) Top: Schematic of a MBA_2_CuCl_4_/(6,5) SWCNT interface and photoexcited electron transfer across the heterojunction interface to modify the channel current of the phototransistor. Bottom: FET device architecture with a back-gated, three-terminal (S, source; D, drain; G, gate) configuration.

Figure 4A shows the linear absorption spectra for (6,5) SWCNT networks, (*R*-MBA)_2_CuCl_4_/(6,5) SWCNT, and (*S*-MBA)_2_CuCl_4_/(6,5) SWCNT heterojunctions. The new peak in the heterojunction structure, labeled X^+^, is assigned to the optical transition of charged excitons (trions) in (6,5) SWCNT networks, based on the original assignment by Matsunaga *et al*.^33^ The presence of the trion peak indicates a ground-state charge carrier density (holes, *vide infra*) in the heterojunctions,^34^ and therefore, ground-state charge transfer occurs at the MBA_2_CuCl_4_/SWCNT interface. The transmission CD measurement in Figure 4B indicates that the MBA_2_CuCl_4_/SWCNT heterojunctions display essentially the same chiroptical response as the neat MBA_2_CuCl_4_ films, suggesting that the SWCNT network does not impact the ability of MBA_2_CuCl_4_ to discriminate between right- and left-handed CPL.

The direction of ground-state charge transfer at the MBA_2_CuCl_4_/SWCNT interface is verified by FET measurements (Figure 4C), by comparing the dark FET transfer curves of the *R*-MBA_2_CuCl_4_/(6,5) SWCNT heterojunction to that of just the (6,5) SWCNTs (inset). Due to the very low conductivity and mobility of chiral MBA_2_CuCl_4_ compared with the SWCNT networks, the channel current of the heterojunction is dominated by hole transport within the (6,5) SWCNT networks. The threshold voltage (*V*_th_) of the heterojunction FET is more than 30 V, a large shift in the positive direction compared to the threshold voltage of the SWCNT FET (*V*_th_ ≈ 10 V). This large shift in *V*_th_ indicates that a net transfer of holes from chiral MBA_2_CuCl_4_ to SWCNTs results in a more p-type SWCNT network. Recent studies suggest that physisorption and redox reaction of Cu^2+^ species on SWCNTs can promote such p-type doping.^35^

Under 405 nm laser illumination, the source–drain current (*I*_DS_) of the heterojunction FET decreases significantly, and the threshold voltage shifts toward the negative direction (Figure 4C,D). Both of these effects are consistent with a photoinduced electron transfer from chiral MBA_2_CuCl_4_ to the SWCNT networks (Figure 4E). Since the (6,5) SWCNT transport channel is heavily p-type for the heterojunction in the dark, the transfer of photoinduced electrons into the SWCNT network compensates some fraction of the native hole density, decreasing *I*_DS_ and generating the negative *V*_th_ shift. Figure 4D displays the photocurrent (*I*_ph_ = *I*_laser_ – *I*_dark_, where *I*_laser_ is the current level of the device under laser illumination and *I*_dark_ is the current level of the device in the dark) for nonpolarized 405 nm laser excitation. No photocurrent is observed for neat MBA_2_CuCl_4_ films (Figure S1).

**Figure 5:**
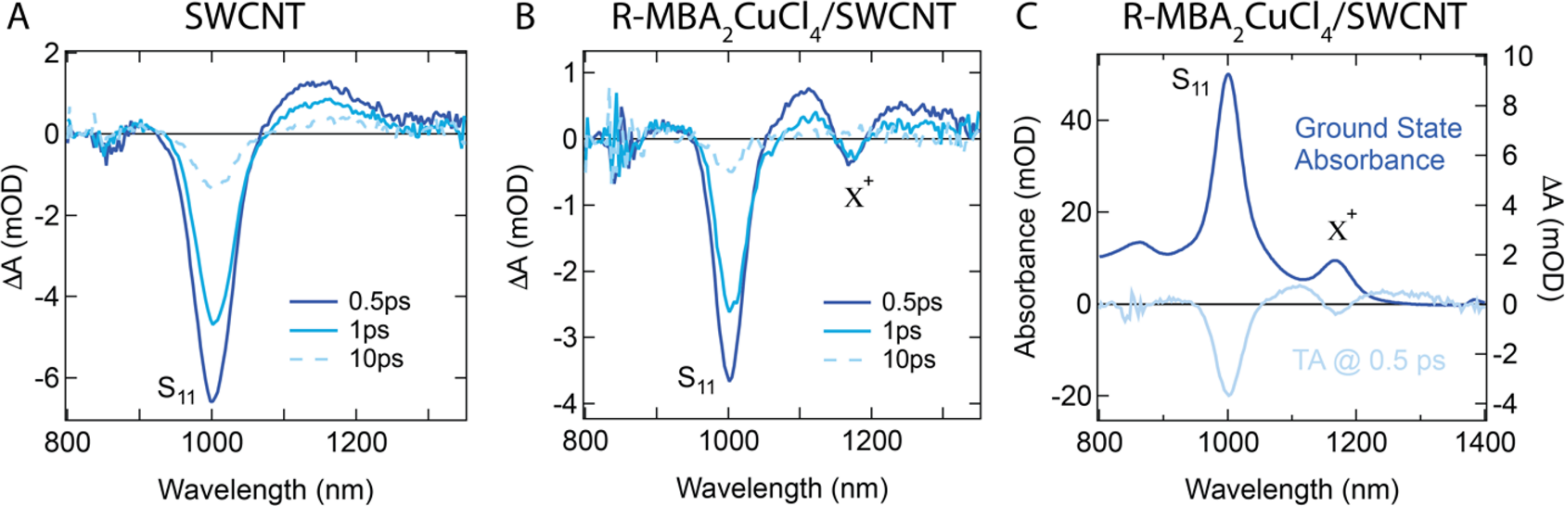
Femtosecond TA spectra of SWCNT (A) and R-MBA_2_CuCl_4_/SWCNT (B) thin films collected in the NIR region (pumped at 400 nm). (C) Comparison of the TA spectrum at 0.5 ps (right axis) to the ground-state absorbance of the R-MBA_2_CuCl_4_/SWCNT (left axis). The scattering background is subtracted from the ground-state absorbance to achieve zero absorbance beyond 1300 nm. The 400 nm pulse energy in the TA experiments is 125 nJ.

The inferred mechanism of photoinduced electron transfer from chiral MBA_2_CuCl_4_ to SWCNT networks is confirmed by transient absorption (TA) spectroscopy. Femtosecond TA spectra of SWCNT (Figure 5A) and MBA_2_CuCl_4_/SWCNT (Figure 5B) thin films are collected in the NIR region using a pump excitation wavelength of 400 nm. The TA spectra of SWCNTs are dominated by the S_11_ exciton bleach at 1000 nm and a small bleach of the K-momentum phonon sideband^36^ at 850 nm, whereas that of MBA_2_CuCl_4_/SWCNT consists of the same bleaches and an additional negative-pointing peak at 1180 nm corresponding to the trion transition. The bleaches of the S_11_ and X^+^ ground states can be seen clearly when compared to the ground-state absorption in Figure 5C. The prompt photoinduced *bleach* of the X^+^ ground-state absorbance suggests a rapid (τ ∼ 0.4 ps; see Figure S2) reduction in the ground-state hole density of the SWCNT network. Thus, this observation is consistent with a photoinduced electron transfer process from MBA_2_CuCl_4_ to SWCNT that compensates some fraction of native holes in the SWCNT network, in agreement with the dark and illuminated FET results discussed above.

### Circularly Polarized Light Detection

**Figure 6:**
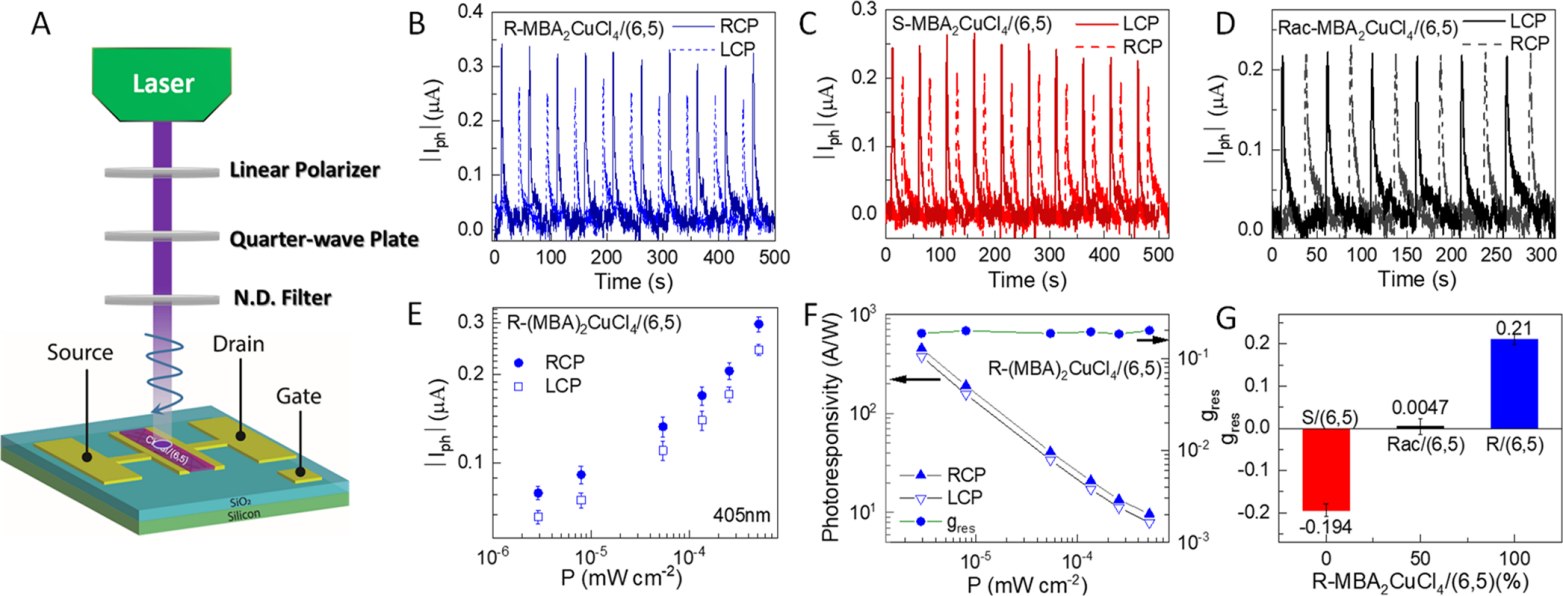
Direct circularly polarized light (CPL) detection using *R*- and *S*-MBA_2_CuCl_4_/(6,5) SWCNT heterojunctions. (A) Schematic showing the experimental setup to generate left (LCP) and right (RCP) circularly polarized light. (B–D) Variation of absolute value of photocurrent change (|*I*_ph_|) *vs* time for *R*-, *S*-, and *rac*- MBA_2_CuCl_4_/(6,5) SWCNT heterojunctions under the illumination of pulse-mode 405 nm RCP and LCP laser separately under *V*_DS_ = 2 V. (E) Photocurrent response as a function of light intensity of (*R*-MBA)_2_CuCl_4_/(6,5) SWCNT heterojunction device under the LCP and RCP illumination, separately. (F) Light-intensity-dependent photoresponsivity and *g*_res_ at *V*_DS_ = 2 V under 405 nm RCP and LCP illumination. (G) Dependence of *g*_res_ factor on composition (expressed as percentage of (*R*-MBA)_2_CuCl_4_), under 405 nm RCP and LCP illumination at *V*_DS_ = 2 V.

Since the CD spectra demonstrate that chiral MBA_2_CuCl_4_/SWCNT heterojunctions can discriminate well between right- and left-handed CPL, we next sought to demonstrate optoelectronic CPL detection in heterojunction photodetectors. The photodetector architecture (Figure 6A) comprises a spin-cast thin film of MBA_2_CuCl_4_ on top of a prefabricated (6,5) SWCNT network that spans the width of a 10 μm bottom-contacted FET transport channel. Continuous-wave left-handed circularly polarized (LCP) and right-handed circularly polarized (RCP) excitation beams were generated using a 405 nm laser diode, a linear polarizer, a quarter-wave plate, and an adjustable neutral density filter. This experimental setup was placed in a nitrogen-filled glovebox and carefully calibrated to ensure equal intensities for the generated LCP and RCP excitation beams.

Figure 6B–D shows the time-dependent photocurrent response from *R*-, *S*-, and *rac*-MBA_2_CuCl_4_/SWCNT heterojunctions, displayed as the absolute value of *I*_ph_, |*I*_ph_|. This experiment utilizes pulsed-mode LCP and RCP illumination with equivalent intensity, a laser pulse duration of 2 s, and a pulse period of 50 s. For the *R*-MBA_2_CuCl_4_/SWCNT heterojunction, |*I*_ph_| under RCP illumination is larger than that under LCP illumination, while the photocurrent anisotropy is reversed for the *S*-MBA_2_CuCl_4_/SWCNT heterojunction. In contrast, there is no photocurrent anisotropy for the *rac*-MBA_2_CuCl_4_/SWCNT heterojunction devices. These results clearly demonstrate that the *R*/*S*-chiral MBA_2_CuCl_4_/SWCNT photodetectors can be used to directly detect CPL. We further evaluated the dependence of photocurrent (|*I*_ph_|) and photoresponsivity (*R*) on illumination intensity (Figure 6E,F). Photoresponsivity (*R*) is defined as
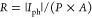
3where *P* is incident light power density and *A* is channel area. The *R*-MBA_2_CuCl_4_/SWCNT heterojunction demonstrates successful discrimination between LCP and RCP 405 nm photons for over 3 orders of magnitude in incident light intensity. Importantly, the photoresponsivity is also much larger than that of previously published 1D/2D perovskite CPL photodetectors, enabling photocurrents that are 4–5 orders of magnitude higher than those of recently published MHS CPL detectors at similar *V*_DS_ = 2 V.^9,11−13^

The anisotropy factor of photoresponsivity (*g*_res_)^28,37^ is calculated to evaluate the effectiveness for discriminating between different photon helicities:
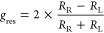
4where *R*_L_ and *R*_R_ are the photoresponsivity under LCP and PCP illumination, respectively. The *g*_res_ magnitude of ∼0.2 is independent of light intensity (Figure 6F) and *V*_DS_ (Figure S3). This intensity independence implies that the photoinduced electron transfer efficiency from *R*-chiral MBA_2_CuCl_4_ to SWCNTs is similarly intensity-independent over this range of light intensities. The average *g*_res_ values of *S*- and *R*-chiral MBA_2_CuCl_4_/SWCNT heterojunctions are about −0.194 and +0.21, respectively (Figure 6G), with a champion *g*_res_ of +0.25 for the *R*-heterojunction (Figure S4). In contrast, the *g*_res_ value of 0.0047 for the (*rac*-MBA)_2_CuCl_4_/SWCNT heterojunction indicates a negligible ability for this heterojunction to discriminate between RCP and LCP photons, in agreement with the negligible CD (Figure 3B).

The large *g*_res_ value obtained here can be compared to recent demonstrations of direct CPL detectors using either chiral MHS^9,11,13,24^ or organic semiconductor^8,10,12,14,38^ active layers, which typically achieve *g*_res_ ≤ 0.1 and require relatively high operating voltages to produce pA–nA currents (Table S1). An exception is the recent report on a chiral 1D MHS CPL detector (in a different vertical architecture) published while we were finalizing this article.^22^ We note that the *current densities* of ∼3 mA/cm^2^ achieved here for our small-area lateral heterojunction devices exceed that report (0.28 mA/cm^2^),^22^ an advantage for the ultimate integration of CPL detector arrays. Importantly, the SWCNT heterojunction approach demonstrated here allows us to achieve large *g*_res_ values, μA currents, and mA/cm^2^ current densities at low operating voltages of *V*_GS_ = 0 V and *V*_DS_ = 2 V (down to 0.01 V for 25 nm thick heterojunctions, *vide infra*) and should be applicable to a broad range of MHS with varying dimensionality and degrees of intrinsic conductivity.

Interestingly, the calculated value of *g*_res_ is 4–5 times higher than the *g*_CD_ value calculated from CD and absorption spectra, where *g*_CD_ factors for *R*- and *S*-MBA_2_CuCl_4_/SWCNT heterojunctions at 405 nm are 0.05 and −0.04, respectively. In a heterojunction active layer such as the ones explored here, the amplification of the absorption anisotropy factor for the chiral absorber layer can arise from two potential sources. First, it is possible that exciton and/or charge carrier diffusion *within the chiral absorber layer* is spin-selective.^17^ In this case, the original anisotropy factor realized by the circular dichroic absorption process could be amplified as excitons or charges moving site to site (*e.g*., by hopping or resonance energy transfer) with a spin-dependent efficiency toward the interface with the charge acceptor. Second, it is possible that the interfacial charge transfer from the chiral absorber layer to the charge acceptor occurs with spin-dependent efficiency. In this case, the original absorption-based anisotropy is amplified by the spin-selective interfacial charge transfer event.^23^ Both of these spin-filtering events represent manifestations of the chiral-induced spin selectivity (CISS) effect, which is hypothesized to arise from the coupling between the magnetic moment of an electron and the effective magnetic field generated by electron propagation through a chiral potential.

**Figure 7:**
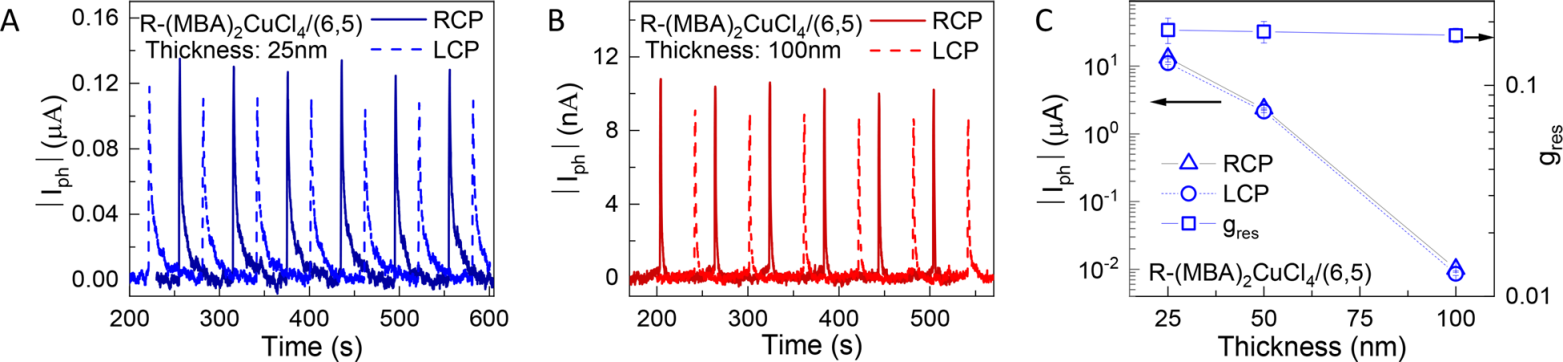
Thickness-dependent CPL detection of R-MBA_2_CuCl_4_/(6,5) SWCNT heterojunctions. (A,B) Time-dependent absolute value of photocurrent change (|*I*_ph_|) of 25 nm (*V*_DS_ = 0.01 V) (A) and 100 nm (*V*_DS_ = 1 V) (B) thick (*R*-MBA)_2_CuCl_4_/SWCNT heterojunctions, separately. (C) |*I*_ph_| and *g*_res_ as a function of thickness of (*R*-MBA)_2_CuCl_4_/SWCNT heterojunctions.

To probe the relevant contributions of spin-selective transport within the MBA_2_CuCl_4_ absorber layer and/or spin-selective interfacial charge transfer at the MBA_2_CuCl_4_/SWCNT interface, we performed a thickness-dependent study. Here, we deposited three different thickness (15, 40, and 90 nm) of the *R*-chiral MBA_2_CuCl_4_ layer on the top surface of identically prepared ∼10 nm thick SWCNT networks (total heterojunction thicknesses of 25, 50, and 100 nm). The time-dependent |*I*_ph_| values of 25 and 100 nm thickness devices are plotted in Figure 7A,B (405 nm, 1 s pulse duration, and 60 s period), while Figure 7C plots the thickness-dependent *I*_ph_ and *g*_res_. The photocurrent magnitude drops off dramatically with increasing thickness, with the 25 nm thick heterojunction showing almost 3 orders of magnitude higher |*I*_ph_| than that of the 100 nm thickness device. Note that the large |*I*_ph_| for the 25 nm device is measured with a very low operating voltage of *V*_DS_ = 0.01 V. Despite the substantial decline in |*I*_ph_| with increasing thickness, there is only a small decline in *g*_res_ (Figure 7C). The calculated *g*_res_ values for 25, 50, and 100 nm thick heterojunctions are ∼0.183, 0.18, and 0.173, respectively. The drop-off of |*I*_ph_| and relatively constant magnitude of *g*_res_ with increasing MBA_2_CuCl_4_ thickness lead us to conclude that spin-selective interfacial charge transfer is the dominant mechanism resulting in amplification of the helicity-dependent anisotropy in these heterojunctions. This result is sensible, given the lack of connectivity between the isolated CuCl_4_ tetrahedra, which leads to inefficient charge/exciton transport within the MBA_2_CuCl_4_ absorber layer. As such, these heterojunctions rely predominantly upon spin-selective interfacial photoinduced electron transfer at the donor/acceptor interface to enable both high photocurrent and a high CPL anisotropy factor.

## Conclusion

In conclusion, we have demonstrated the direct detection of circularly polarized light using a nanoscale heterojunction composed of organic/inorganic hybrid chiral semiconductors: MBA_2_CuCl_4_ and highly enriched semiconducting SWCNT networks. On one hand, the very high photoresponsivity (452 A/W), high anisotropy factor (0.21 average, 0.25 champion), low operating voltage (0.01 V), scalability, and CMOS compatibility of our CPL photodetector makes it promising for a broad range of direct CPL detection applications. On the other hand, the use of a charge-separating heterojunction establishes a platform for exploring spin-selective charge carrier transport and transfer in hybrid chiral systems. These strategies can be applied to a broad range of emerging chiral hybrids for both fundamental studies and high-performance optoelectronic devices. The tunable dimensionality and optical, electronic, and spin degrees of freedom for both donor and acceptor imply that a wide range of emergent phenomena and optoelectronic devices can be realized by such heterojunctions.

## Methods

### Materials

All chemicals were used as received unless otherwise indicated. Phenethylamine (PEA), (*R*)-(+)-α-methylbenzylamine (*R*-MBA, 98%, ee 96%), (*S*)-(−)-α-methylbenzylamine, (*S*-MBA, 98%, ee 98%), (±)-α-methylbenzylamine (*rac*-MBA, 99%), copper(II) chloride dihydrate (CuCl_2_·2H_2_O, 99.999%), hydrochloric acid (ACS reagent, 37 wt % in water), and *N*,*N*-anhydrous DMF were purchased from Sigma-Aldrich.

### Synthesis of Phenethylammonium Chloride (PEACl)

Five milliliters of PEA and 15 mL of ethanol were added to a 250 mL round-bottom flask. The mixture was stirred at 0 °C using an ice–water bath, followed by adding 10 mL of HCl (37 wt % in water) dropwise. The solution was stirred for 2 h. Subsequently, the solvent was removed by a rotatory evaporator. The white powder was then recrystallized from ethanol/diethyl ether, generating PEACl.

### Synthesis of Methylbenzylammonium Chloride (MBACl)

The synthesis of MBACl is similar to that of PEACl. Briefly, 5 mL of methylbenzylamine (*R-/S-/rac*-MBA) and 15 mL of ethanol were added to a 250 mL round-bottom flask. The mixture was stirred at 0 °C using an ice–water bath. Ten milliliters of HCl (37 wt % in water) was then added to the mixture dropwise. The solution was stirred for 2 h. Subsequently, the solvent was removed by a rotatory evaporator. The white powder was then recrystallized from of mixture of ethanol/diethyl ether, generating MBACl.

### Synthesis of PEA_2_CuCl_4_ Single Srystals

First, 171 mg of CuCl_2_·2H_2_O (1 mmol), 315 mg of PEACl (2 mmol), and 6 mL of methanol were loaded in a glass vial. The mixture was then stirred under heat until all solids were dissolved, yielding a clear dark green solution. The vial was then allowed to cool to room temperature, and dark-yellow plate-like crystals (PEA_2_CuCl_4_) were precipitated out of the solution. These crystals were then filtered, washed with diethyl ether, and dried in a vacuum overnight.

### Synthesis of (*R*-/*S*-/*rac*-MBA)_2_CuCl_4_ Single Crystals

The synthesis of MBA_2_CuCl_4_ is very similar to that of PEA_2_CuCl_4_. First, 171 mg of CuCl_2_·2H_2_O (1 mmol), 315 mg of MBACl (2 mmol), and 2 mL of isopropyl alcohol were loaded in a glass vial. The mixture was then stirred under heat until all solids were dissolved, yielding a clear dark green solution. The vial was then allowed to cool to room temperature, and green plate-like crystals (MBA_2_CuCl_4_) were precipitated out of the solution. These crystals were then filtered, washed by diethyl ether, and dried in vacuum overnight. Larger MBA_2_CuCl_4_ crystals (1 cm × 1 cm) can be grown by slowly diffusing diethyl ether into a methanol solution of MBA_2_CuCl_4_.

### Preparation of PEA_2_CuCl_4_ and (*R*-/*S*-/*rac*-MBA)_2_CuCl_4_ Thin Films

Glass or quartz substrates were washed sequentially using acetone and isopropyl alcohol in a sonicator for 100 min each, followed by an ultraviolet-ozone treatment for 15 min. Precursor solutions were prepared by dissolving crystals in DMF with a mass-to-volume ratio of 10 wt % (*e.g.*, 20 mg in 200 μL). Thin films were prepared by spin-coating the corresponding precursor solution onto substrates using a spin rate of 4000 rpm for 30 s, followed by thermal annealing at 100 °C for 10 min. Thin films on glass substrates were used for XRD measurements. Thin films on quartz substrates were used for linear optical and CD measurements.

### Single-Crystal X-ray Diffraction

Single-crystal full-sphere data were collected using a Bruker KAPPA APEX II diffractometer equipped with an APEX II CCD detector using a TRIUMPH monochromator with a Mo Kα source (λ = 0.71073 Å) with MX Optics or a Bruker D8 VENTURE diffractometer equipped with a Kappa goniometer stage, a PHOTON II CPAD detector, and an IμS 3.0 Mo Kα source (λ = 0.71073 Å). Data were collected at 293 K. The collected data were integrated and applied with multiscan absorption correction using the APEX2 or APEX3 software. Structure solution was obtained by direct methods using the SHELXS program and refined using the least-squares method by employing the SHELXL program within the Olex2 software.^39^

### Linear Optical Absorption Measurements

For powder absorption, powder samples were first obtained by grinding single crystals using a mortar and pestle. Linear optical absorption spectra were obtained by performing optical diffuse reflectance measurements in a Cary 5000 UV–vis–NIR spectrometer operating in the 1500–300 nm region at room temperature. BaSO_4_ was used as the reference for 100% reflectance, and BaSO_4_ was also used to dilute powder samples for all measurements. Linear optical absorption spectra of powders were generated by converting reflectance to absorption data using the Kubelka–Munk equation: α/*S* = (1 – *R*)^2^/(2*R*), where *R* is the reflectance and α and *S* are the absorption and scattering coefficients, respectively. For thin film absorption, absorption spectra were collected in the transmission mode using a quartz substrate as the reference of 100% reflectance.

### CD Measurements

CD measurements were performed using a Jasco J-715 spectropolarimeter with the thin film placed in the beam path. The obtained spectra were averages of 3–5 scans. The CD spectra were collected in the 200–600 nm range with 0.2 nm resolution.

### Transient Absorption Measurements

Transient absorption measurements were performed using a Coherent Libra Ti:sapphire laser, with an output of 800 nm at 1 kHz. The 800 nm beam was directed into a TOPAS optical parametric amplifier to generate a pump pulse (∼150 fs) and was modulated at 500 Hz through an optical chopper to block every other laser pulse. Femtosecond TA spectra were collected using a Helios spectrometer (Ultrafast Systems). A small amount of 800 nm light was used to pump a 1 cm thick sapphire crystal to generate 750–1500 nm probe light for NIR TA. All samples were prepared under a N_2_ atmosphere and measured using air-free holders.

### Preparation of Polymer and s-SWCNT Dispersions

SWCNT dispersions and inks were prepared as previously reported.^34^ The SWCNT raw material was the CoMoCAT SG65i material, commercially obtained from Chasm. The polymer used in this study was poly[(9,9-dioctylfluorenyl-2,7-diyl)-*alt-co*-(6,6′-[2,2′-bipyridine])] (PFO-BPy), purchased from American Dye Source. When used to disperse the CoMoCAT SG65i material, PFO-BPy preferentially disperses the (6,5) semiconducting SWCNT and lowers the metallic SWNT purity level down to less than 1% in the SWCNT dispersion. To prepare the SWCNT/polymer mixture, 7.5 mg of the SWCNT material and 30 mg of PFO-BPy were loaded into 15 mL of toluene. This dispersion was sonicated using a tip sonicator (Cole-Parmer CPX 750) for 30 min, with a 0.5 in. diameter tip at 40% power. After this initial sonication step, the dispersion was centrifuged for 5 min on a Beckman optimaTM L-100XP ultracentrifuge in an SW32 rotor at a centrifugal force of 13,200 rpm. The (6,5)-enriched supernatant was collected by pipet, and the remaining solid pellet was discarded. After multiple (6,5) dispersions (6–10) were collected, the majority of the excess PFO-BPy polymer was removed through three continuous 20 h ultracentrifuge runs at a centrifugal force of 24,100 rpm. After each 20 h centrifuge run, the polymer-rich supernatant was decanted and discarded, while the (6,5)-enriched SWCNT pellet was collected and redispersed into toluene. After the end of the third polymer removal run, the SWCNT pellet was redispersed in toluene for further spray-coating.

### Preparation of s-SWCNT Networks

s-SWCNT networks were prepared by spraying the prepared s-SWCNT inks onto SiO_2_/Si wafers or quartz substrates using an ultrasonic sprayer with a Sonotek 120 kHz impact nozzle at room temperature.^34^ Before deposition, the substrates were rinsed with acetone and isopropyl alcohol and treated with UV-ozone plasma for 10 min. The SWCNT dispersion was delivered to the ultrasonic nozzle using a syringe pump at 300 μL/min, and a nitrogen jet (7 std L/min) deflected the atomized ink to the substrate. The power delivered to the nozzle was 0.8 W. The substrate was heated up to 130 °C to vaporize the toluene solvent as the ink droplets impinged upon the substrate. After spray coating, the s-SWCNT thin film was soaked in a 78 °C toluene bath for 10 min to remove excess fluorene-based polymers.

### Preparation of MBA_2_CuCl_4_/SWCNT Heterojunction

Different MBA_2_CuCl_4_/DMF solutions with various concentrations (5, 10, and 20 wt %) were first prepared. The MBA_2_CuCl_4_/DMF was then spin-coated on top of the patterned SWCNT network using a spin rate of 4000 rpm for 30 s, followed by thermal annealing at 100 °C for 10 min.

### Device Fabrication

The typical FET device was fabricated on a 200 nm thick SiO_2_/highly doped p-type Si wafer (1–10 Ω cm^–1^) purchased from MTI Corporation using the standard optical lithography technique. Gold electrodes with a titanium adhesion layer (5 nm thick Ti/20 nm thick Au) were deposited on the patterned substrate using the thermal evaporation deposition system, and the gate electrode was directly contacted with a highly doped Si wafer. All of these procedures were performed in the cleanroom. As designed, the channel lengths (*L*_ch_) of the devices used in this study were ∼10 μm, and the channel widths (*W*_ch_) were ∼1000 μm.

### Photocurrent Response Measurement

All photocurrent measurements were performed inside a nitrogen atmosphere glovebox with 3 mbar pressure. This type of measurement was conducted using two Keithley 2400 source meters: one was used to supply the power to the laser diode, and the other was used to supply the voltage to devices and monitor the channel current. All of the experimental parameters and data were controlled and collected by a self-developed LabVIEW program. The 405 nm laser illumination was supplied using KOKUYO laser diodes, and the linear polarizer and 405 nm quarter wave plate were purchased from Thorlabs Inc. The power density of the laser diode was adjusted by inserting a series of neutral density filters between the quarter-wave plate and the device, and the power of the laser was calibrated using a Field Mate laser power meter from Coherent Inc. The pulse-mode laser signal was generated using an Agilent 33220A function generator with controlled software to power the laser diode, and the energy of the pulse-mode laser was calibrated using Ophir Laserstar P/N 7021600 power meter with a lower limit of detection of 1 pW.

## Supporting Information Available

Current–voltage plot for *R*-MBA_2_CuCl_4_ device in dark and light; transient absorption analysis of electron transfer time in *R*-MBA_2_CuCl_4_/SWCNT heterojunction; dependence of *g*_res_ anisotropy factor on *V*_DS_ for (*R*-MBA)_2_CuCl_4_/SWCNT heterojunction device; variation of *g*_res_ anisotropy factor for 10 (*R*-MBA)_2_CuCl_4_/SWCNT heterojunction devices; crystal data and structure refinement for (*R*-MBA)_2_CuCl_4_, (*S*-MBA)_2_CuCl_4_, and (*rac*-MBA)_2_CuCl_4_; summary of *g*_CD_ from recent literature; summary of CPL detector performance metrics from literature studies using in-plane two- or three-terminal device configurations (PDF)
