# Supplementary material for: Direct Detection of Circularly Polarized Light Using Chiral Copper Chloride–Carbon Nanotube Heterostructures
Source: ACS Nano. 2021 Apr 6;15(4):7608–17. doi: 10.1021/acsnano.1c01134 (PMC10156083; doi:10.1021/acsnano.1c01134)
Supplement: Supplementary file 1 — nn1c01134_si_001.pdf [file nn1c01134_si_001.pdf]

Supporting information

Direct Detection of Circularly Polarized Light Using Chiral Copper Chloride-Carbon Nanotube Heterostructures

Ji Hao,[1†] Haipeng Lu,[*1,2†] Lingling Mao,[3] Xihan Chen,[1] Matthew C. Beard,[1] and Jeffrey L. Blackburn[*1]

[1] Chemistry & Nanoscience Center, National Renewable Energy Laboratory, Golden, Colorado 80401, United States

[2] Department of Chemistry, The Hong Kong University of Science and Technology, Clear Water Bay, Kowloon, Hong Kong, China (SAR)

[3] Materials Department and Materials Research Laboratory University of California, Santa Barbara, California 93106, United States

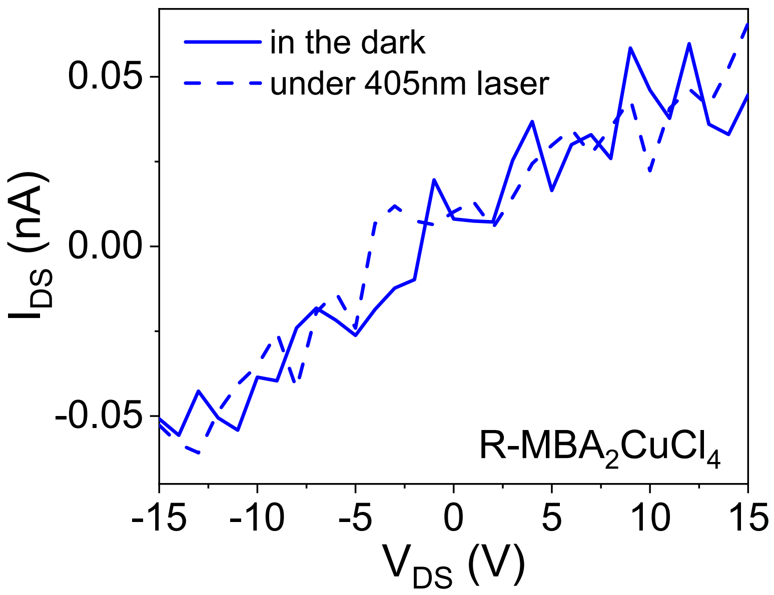

Figure S1: Current to voltage sweeping plot of $R-MBA_2CuCl_4$ device (L_{ch}=10μm) in the dark and under the 405nm wavelength laser.

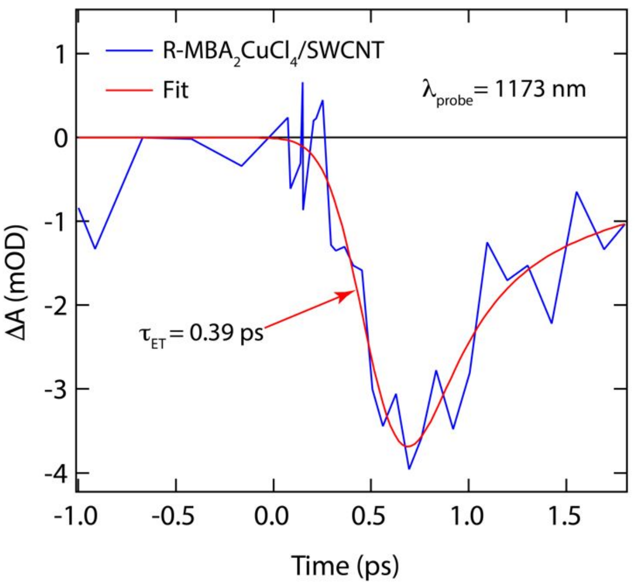

Figure S2: TA dynamics of the X^+ ground-state bleach, probed at 1173 nm after pumping at 400 nm. Raw data shown as the blue trace and a fit is shown as the red trace. The fit consists of an exponential rise time convolved with an exponential decay. The rise time extracted from this fit is 0.39 ps, which should represent the electron transfer time (τ_{ET}).

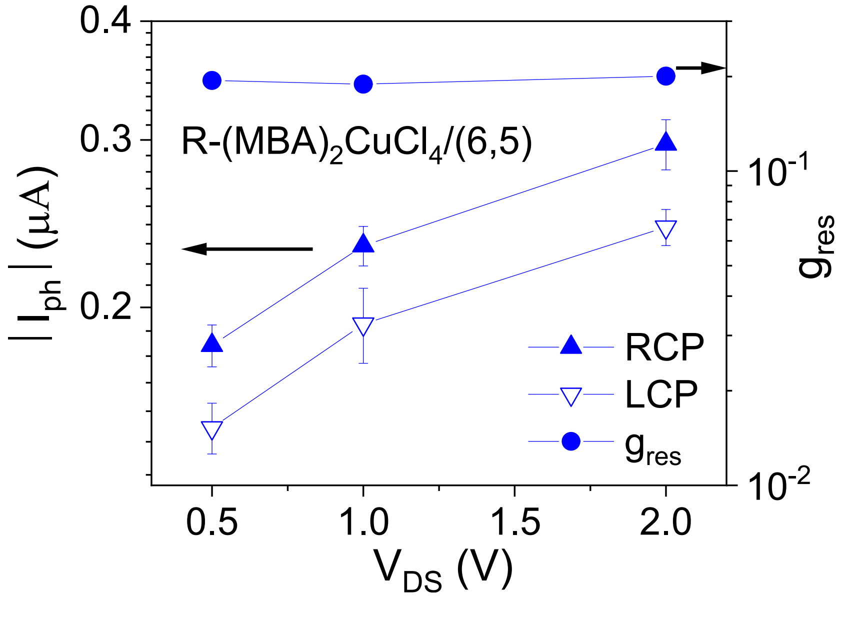

Figure S3: Source-drain voltage dependent photocurrent response and anisotropy factor (g_{res}) of (R-MBA)$_2$CuCl$_4$/(6,5) SWCNT heterojunction device under the RCP and LCP light.

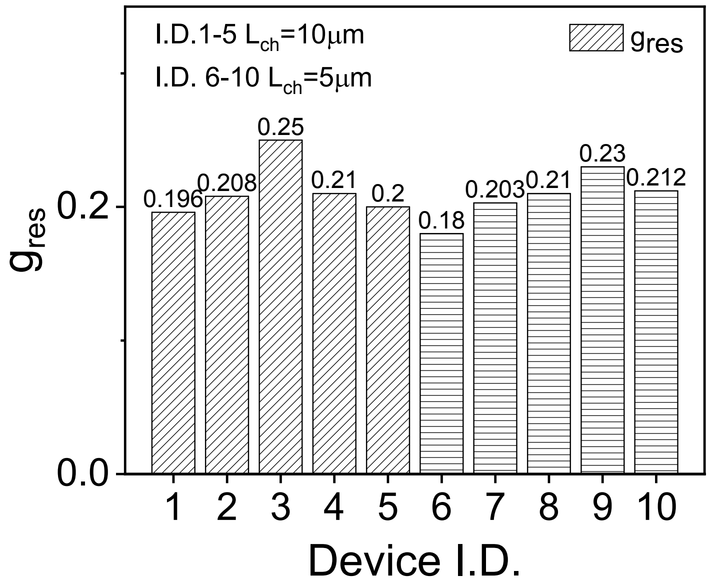

Figure S3: Variation of anisotropy factors (g_{res}) of 10 different $(R\text{-}MBA)_2CuCl_4$/(6,5) SWCNT heterojunction devices with 50nm thickness under 2V voltage bias.

Table S1. Crystal Data and Structure Refinement for (*R*-MBA)$_2$CuCl$_4$, (*S*-MBA)$_2$CuCl$_4$, and (*rac*-MBA)$_2$CuCl$_4$

	Compound Name		
	(*R*-MBA)$_2$CuCl$_4$	(*S*-MBA)$_2$CuCl$_4$	(*rac*-MBA)$_2$CuCl$_4$
Empirical formula	$C_{16}H_{24}N_2Cl_4Cu$	$C_{16}H_{24}N_2Cl_4Cu$	$C_{16}H_{24}N_2Cl_4Cu$
Formula weight		449.71	
Temperature		293(2) K	
Wavelength		0.71073 Å	
Crystal system	Monoclinic	Monoclinic	Orthorhombic
Space group	*C2*	*C2*	*Aea2*
Unit cell dimensions	a = 10.580(7) Å, α = 90°<br>b = 7.235(3) Å, β = 95.96(4)°<br>c = 13.974(10) Å, γ = 90°	a = 10.542(10) Å, α = 90°<br>b = 7.259(7) Å, β = 95.560(19)°<br>c = 13.941(13) Å, γ = 90°	a = 27.6799(8) Å, α = 90°<br>b = 10.5667(4) Å, β = 90°<br>c = 7.2446(2) Å, γ = 90°
Volume	1063.9(11) $Å^3$	1061.8(18) $Å^3$	2118.94(12) $Å^3$
Z	2	2	5
Density (calculated)	1.404 g/cm^3	1.407 g/cm^3	1.762 g/cm^3
Absorption coefficient	1.528 mm^{-1}	1.531 mm^{-1}	1.918 mm^{-1}
F(000)	462	462	1155
θ range for data collection	1.465 to 31.230°	1.468 to 30.416°	1.471 to 26.366°
Index ranges	-15<=h<=15, -10<=k<=10, -20<=l<=15	-14<=h<=14, -9<=k<=10, -19<=l<=18	-34<=h<=33, -13<=k<=13, -9<=l<=8
Reflections collected	6593	4237	12646
Independent reflections	3265 [R_{int} = 0.0324]	2725 [R_{int} = 0.0290]	2140 [R_{int} = 0.0299]
Completeness to θ = 25.242°	100%	97.3%	100%
Refinement method		Full-matrix least-squares on F^2	
Data / restraints / parameters	3265 / 1 / 107	2725 / 1 / 107	2140 / 1 / 107
Goodness-of-fit	0.941	0.917	1.128
Final R indices [I > 2σ(I)]	R_{obs} = 0.0355, wR_{obs} = 0.0717	R_{obs} = 0.0321, wR_{obs} = 0.0592	R_{obs} = 0.0256, wR_{obs} = 0.0675
R indices [all data]	R_{all} = 0.0671, wR_{all} = 0.0820	R_{all} = 0.0555, wR_{all} = 0.0659	R_{all} = 0.0274, wR_{all} = 0.0685
Largest diff. peak and hole	0.380 and -0.291 $e·Å^{-3}$	0.379 and -0.209 $e·Å^{-3}$	0.249 and -0.170 $e·Å^{-3}$

$R = \Sigma||F_o|-|F_c|| / \Sigma|F_o|$, $wR = \{\Sigma[w(|F_o|^2 - |F_c|^2)^2] / \Sigma[w(|F_o|^4)]\}^{1/2}$ and $w=1/[\sigma^2(Fo^2)+(0.0297P)^2]$ where $P=(Fo^2+2Fc^2)/3$

Table S2. Summary of g_{CD} from literature reports and our paper.

Compound	g_{CD}	Reference
(R-/S-MBA)$_2$PbI$_4$	0.0012	Ref. 17
(R-/S-MBA)$_2$SnI$_4$	0.0013	Ref. 18
(R-/S-MBA)PbI$_3$	0.02	Ref. 9
(R-/S-NEA)PbI$_3$	0.04	Ref. 22
**R-MBA$_2$CuCl$_4$**	**0.1**	**Our work**

Table S3. Summary of chiral molecules' CPL detector by using in-plane two- or three-terminal device configuration.

	Chiral Materials	Max g_{res} factor	Applied voltage (V)	Signal current level	Device Dimension	Reference:
1	R/S-(α-PEA)PbI_3 single crystal	0.1 (395nm)	V_{DS} = 20V	10^{-12}A	L_{ch}=10 μm<br>W_{ch}=500 μm	Ref. 9
2	Quasi-2D, [(R)-b-MPA]2$MAPb_2I_7$ and[(S)-b-MPA$]_2MAPb_2I_7$	0.11~0.20 (532nm)	V_{DS} = 10V	10^{-9}A	L_{ch}=50 μm<br>W_{ch}=1000 μm	Ref. 13
3	Quasi-2D crystalline perylene diimides (PDIs)<br>R-ClCPDI-Ph-CF	0.12 (495nm)	V_{DS} = 10V<br>V_{GS} = 10 V	10^{-8}~10^{-9} A	NA	Ref. 12
4	Chiral binaphtyl hybrid with P3CT polymer	0.1 (375 nm)	V_{DS} = 5 V	10^{-8}~10^{-9} A	L_{ch}=90 μm<br>W_{ch}=1000 μm	Ref. 10
5	Chiral PDIs	~0.057 (635 nm)	V_{DS} = 80V<br>V_{GS} = 40 V	10^{-10} – 10^{-9} A	L_{ch}=50 μm<br>W_{ch}=1000 μm	Ref. 14
	Chiral PDIs	~0.01 (730 nm)	V_{DS} = 80V<br>V_{GS} = 40 V	10^{-10} – 10^{-9} A	L_{ch}=50 μm<br>W_{ch}=1000 μm	Ref. 14
6	R-β-MPA]4AgBiI8 ((R)-β-MPA	0.22 (R) (520nm)	V_{DS}=5V	10^{-9} A – 10^{-8} A	0.32mm^2	Ref. 24
7a	S/R-MBA_2CuCl_4 /(6,5) SWCNTs	0.21~0.25 (405nm)	V_{DS} = 2V<br>V_{GS} = 0 V	~10^{-6} A	L_{ch}=10 μm<br>W_{ch}=1000 μm<br>t = 50 nm*	Our work
7b	S/R-MBA_2CuCl_4 /(6,5) SWCNTs	0.20 (405nm)	V_{DS} = 0.01 V<br>V_{GS} = 0 V	~10^{-6} A	L_{ch}=10 μm<br>W_{ch}=1000 μm<br>t = 25 nm*	Our work

* Total film thickness of SWCNT film + MBA_2CuCl_4 film thickness
